# Gut microbiota profiling variated during colorectal cancer development in mouse

**DOI:** 10.1186/s12864-022-09008-3

**Published:** 2022-12-22

**Authors:** Jingjing Liu, Wei Dong, Jian Zhao, Jing Wu, Jinqiang Xia, Shaofei Xie, Xiaofeng Song

**Affiliations:** 1 Department of Biomedical Engineering, Nanjing University of Aeronautics and Astronautics, Nanjing, 210016 China; 2 The State Key Laboratory of Translational Medicine and Innovative Drug Development, Jiangsu Simcere pharmaceutical Co., Ltd, Nanjing, 210016 China; 3 School of Biomedical Engineering and Informatics, Nanjing Medical University, Nanjing, 211166 Jiangsu China

**Keywords:** Colorectal inflammation, Colorectal cancer, Gut microbiota, 16S rRNA

## Abstract

**Background:**

The imbalance of intestinal flora may promote the occurrence and development of colorectal cancer, changes of the intestinal flora during the development of colorectal cancer and the mechanism that promotes the colorectal cancer were discovered in this study. Deep sequencing of the microbial 16 s ribosomal RNA gene was used to investigate alterations in feces samples of mice at the early inflammation stage and fully developed stage of colorectal cancer.

**Results:**

According to PCoA analysis and ANOSIM test, we found the intestinal flora had significantly changed in mice with colorectal inflammation or colorectal cancer compared with healthy mice (*p* < 0.05). Using correlation analysis, we found that *Muribaculaceae* and *Bacteroidaceae* had strong excluding interactions. The functional changes of the gut microbiota include the up-regulation of the cancers pathway and the down-regulation of the replication and repair pathways.

**Conclusion:**

Our study found the intestinal flora of mice suffering from colorectal inflammation and colorectal cancer has changed significantly, especially the decrease of *Muribaculaceae* and the increase of *Bacteroidaceae*. We suppose that these two floras may play an important role in development of colorectal cancer.

**Supplementary Information:**

The online version contains supplementary material available at 10.1186/s12864-022-09008-3.

## Introduction

Colorectal cancer (CRC) is the fourth-leading cause of cancer-related deaths worldwide [1]. Both environmental and genetic factors contributed to colorectal cancer development. The gut microbiota widely inhabited in intestine and has been proved to have a constant crosstalk with the intestinal epithelium [2]. The micro-organisms involved in various host’s biological functions, such as energy metabolism and immune maturation. They are important for gastrointestinal physiology, and changes in their abundance and composition can break the ecological balance of the gastrointestinal tract, leading to intestinal diseases. Gut microbiota has been widely studied in recent years, its role in colorectal carcinogenesis has been investigated. It is reported that transferring stool from CRC patients into germ-free mice could promote tumour formation in conventional mice given azoxymethane to induce colon neoplasia [3]. A number of 16S ribosomal RNA (rRNA) or shotgun sequencing studies have been used to characterize the CRC microbiota in animal and human fecal samples. Overall, compared with the microbiota of healthy individuals, global compositional shift occurred in CRC microbiota reflecting a different ecological microenvironment. Generally, CRC microbiota has a higher species richness, lower abundance of probiotics, and increased abundance of harmful bacteria [4]. These studies point out the functional importance of gut microbiota on carcinogenesis, and provide evidence for a set of microorganisms that might cause CRC.

Chronic inflammation is a crucial risk factor for CRC, the inflammatory state of colon strongly influenced the development of CRC [5]. Severe inflammation of the colon increases the likelihood of developing CRC in patients. Several inflammation-associated genes such as cyclooxygenase-2 (COX-2), nitric oxide (NO), nitric oxide synthase 2 (NOS-2), and the interferon-inducible gene 1-8 U are increased in inflamed mucosa and remain elevated in colonic neoplasms [6–8]. Germ-free mice implanted of stool from CRC patients has increased histological inflammation and expression of inflammatory gene markers [9, 10]. *F. nucleatum* could generate a pro-inflammatory environment for colorectal neoplasia progression in ApcMin mice [11]. During the CRC development, the inflammation was in a dynamic change. Inflammation is closely associated with CRC, and is the inducer and early symptom of CRC.

The gut microbiota was proved to be stable in health individuals over a long period [12], however, according to Liang, Xujun, et al.’s work, there were longitudinal shifts in the microbial community that occur with colitis-associated colorectal cancer. Description of the phylotype profiles provided a comprehensive view of the dynamic changes to the gut microbiota that occur in colitis-associated cancer [13]. Inflammation exists and changes dynamically during the CRC development. The profiles of gut microbiota in inflammation will help to understand the pathological process of CRC. However, there were few studies focus on the profile of gut microbiota in inflammation stage during CRC development. Depicting the micro-organisms community at both inflammation and tumorigenesis periods of CRC will further reveal the interactions between gut microbiota and host, and provide new strategies for CRC prevention and treatment.

In this study, we simulated the inflammatory microenvironment for CRC development in mice by intraperitoneal injection of azoxymethane (AOM) and adding dextran sulfate sodium salt (DSS) to their drinking water. Tumor and necrocytosis were observed in intestine by animal dissections and cell hematoxylin-eosin staining. Feces samples over the CRC development were collected and the microbial community was studied. The species richness and diversity of gut microbiota were firstly decreased and ultimately increased during the CRC development. The composition of gut microbiota had significantly changed at the CRC initial formation period (inflammation) and CRC fully generated period. Significantly changed bacteria and their internal interactions were uncovered. The mouse model we used in this study mimic the inflammatory environment that could induce CRC in human. With restrained genomic and habitat environmental factors, we were able to focus on the study of inflammation-induced CRC and its impact on gut microbiota, as well as the interaction between microbiota and host. To our knowledge, this was the first study in mouse that characterize the gut microbiota community structure in phylum, family and genus levels at different inflammation-induced CRC developing periods.

## Results

### Inflammation evaluation and colorectal tissue staining analysis

**Fig. 1:**
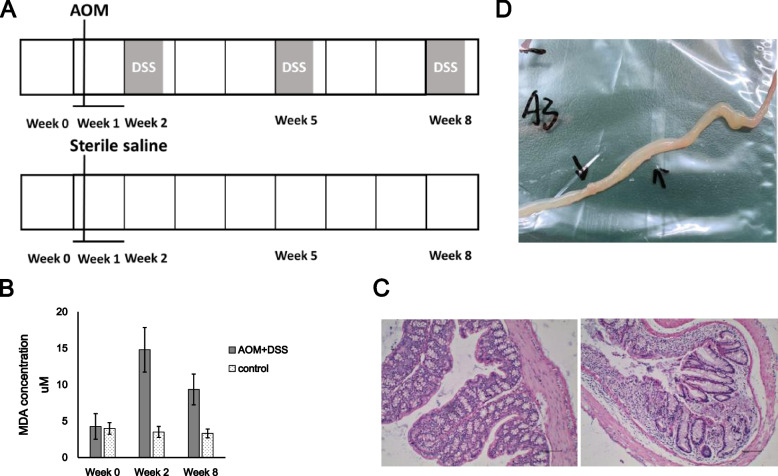
Establishment of mouse colorectal cancer model and biochemical testing. **A** Timeline of animal administration in control (bottom) and experimental (top) mice. **B** Inflammation level in control and experimental mice at week 0, 2 and 8. **C** HE staining of colorectal tissue in control (left) and experimental (right) mice. **D** Macroscopic colorectal tumors in experimental mice

AOM and DSS were used in order to cause inflammation and lead to CRC ultimately. The AOM and DSS administration cycle was illustrated in Fig. 1A. Serum samples from week 0, 2 and 8 were collected and submitted for inflammation assessment using MDA Assay Kit. The result showed that after AOM injection and 1 week’s DSS intake, the mice undergo sever inflammation. While the inflammation tends to mitigate and maintained at a less sever state in tumorigenesis period of CRC (Fig. 1B). CRC was verified by tissue dissection and HE staining. Cell necrosis occurred in colorectal tissue in experimental mice (Fig. 1C). Meanwhile, tumors were observed in the surface of colorectal tissue in experimental mice (Fig. 1D).

### Overview of the 16S rRNA sequence data

FastQC analysis showed that the read length and percentage of GC content are as expected, per base sequence quality plot showed high quality scores at each position in the read across all reads (Additional file 1 fig. S1). The rarefaction curve showed that the sequencing depth is enough for species identification (Additional file 1 fig. S2). Totally, 246,018 16S rRNA reads from 12 samples were obtained. In different groups, the average number of reads per sample in group BC, group C, group L, and group H were 21,863, 21,317, 20,998, and 19,189. We generated OTUs at 97% similarity level and the total number of OTUs was 2371, the OTUs number of each sample was shown in Table 1. α diversity and β diversity of gut microbiota between group C and BC were compared in order to prove the stability of gut microbial community composition during the mice growth (Additional file 1 table S1, Additional file 1 fig. S3 and Additional file 1 fig. S4). No significant variation was observed between group C and BC. Thus, gut microbiota community was similar in health individual mouse and was stable during the mouse growth. Therefore, we use fecal samples in group C as controls for both group L and group H.

**Table 1:** Statistics of sequencing and OTUs of 16S rRNA. C: Group C, L: Group L, H: Group H

SampleID	Number of Reads	Observed OTUs
**C1**	**18,026**	**596**
**C2**	**24,602**	**776**
**C3**	**23,776**	**741**
**C4**	**18,864**	**378**
**L1**	**20,871**	**505**
**L2**	**18,748**	**810**
**L3**	**23,866**	**437**
**L4**	**20,509**	**458**
**H1**	**21,569**	**544**
**H2**	**22,877**	**628**
**H3**	**16,734**	**706**
**H4**	**15,576**	**642**

**Fig. 2:**
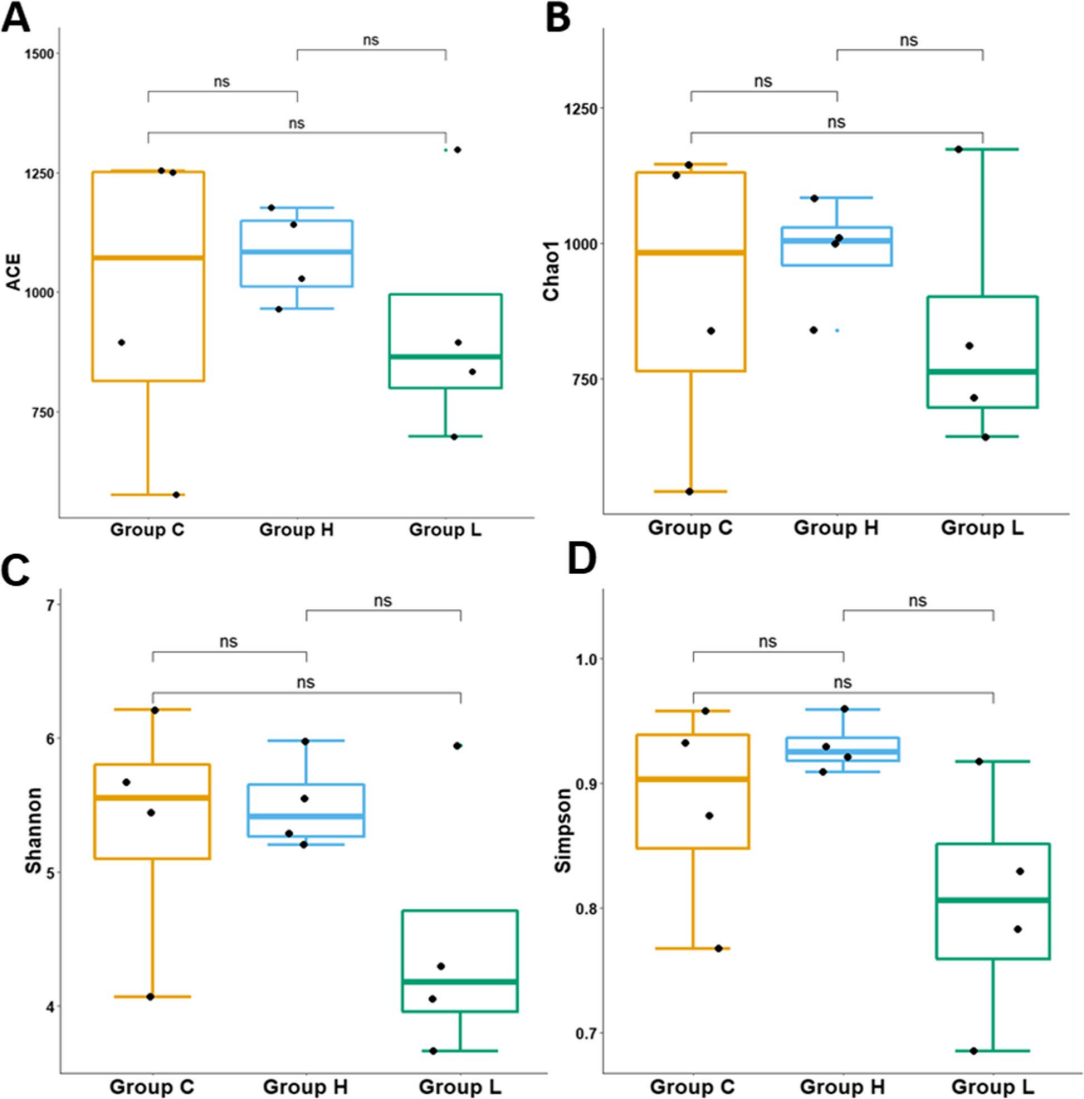
Microbial α diversity in feces samples of Group C, Group L and Group H. **A** Boxplots of ACE Index. **B** Boxplots of Chao1 Richness Index. **C** Boxplots of Shannon Diversity Index. **D** Boxplots of Simpson Diversity Index. ns: *p* > 0.05, no significance; *: *p* < = 0.05; **: *p* < = 0.01

**Fig. 3:**
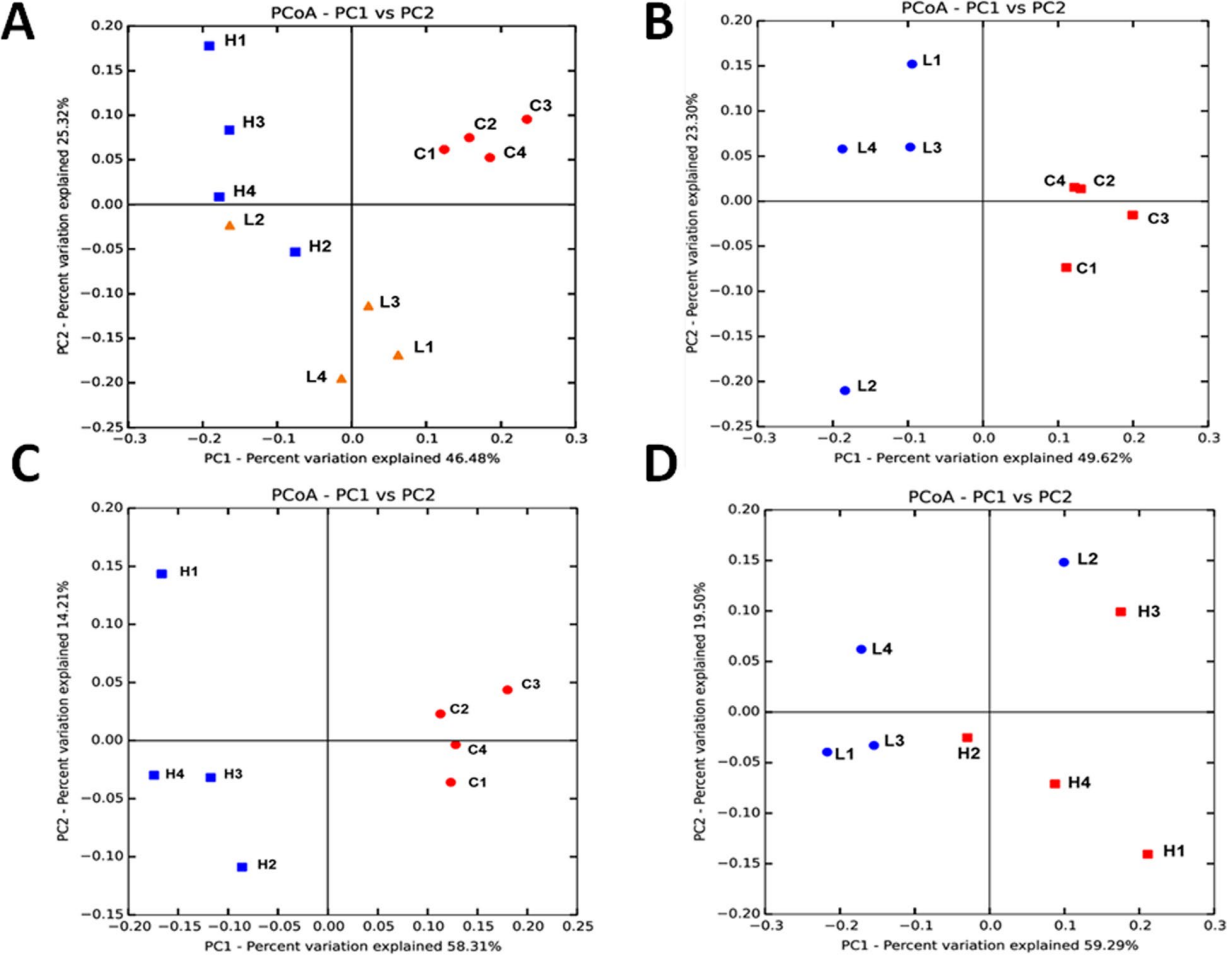
Microbial β diversity in feces samples of Group C, Group L and Group H. **A** PCoA plot of Group C, Group L and Group H, showed a significant difference between health, colorectal inflammation and colorectal cancer. **B** PCoA plot of Group C and Group L, showed a significant difference before and after mice got colorectal inflammation. **C** PCoA plot of Group C and Group H, showed a significant difference before and after mice got colorectal cancer. **D** PCoA plot of Group L and Group H, showed no significant change between mice got colorectal inflammation and mice got colorectal cancer in their intestinal flora

ACE Index, Chao1 Index, Shannon Index, and Simpson Index were used to describe the α diversity of the gut microbiota in different stage of colorectal cancer (Fig. 2). The average ACE Index of group C, group L, and group H were 993.1, 930.6, and 1077.0. The average Chao1 Index of group C, group L, and group H were 912.3, 835.0, and 982.5. During the colorectal cancer development, the species richness of gut microbiota was initially decreased and ultimately increased. The average Shannon Index for group C, group L, and group H were 5.35, 4.49, and 5.50, the average Simpson Index for group C, group L, and group H were 0.88, 0.80, and 0.93, group H has the highest diversity among the three groups. During the colorectal cancer development, the diversity of gut microbiota was initially decreased and ultimately increased. Then welch’s t-test was used to identify significant differences of α-diversity between different groups. However, the results showed that there were no significant differences between their α-diversity. Principal coordinate analysis (PCoA) based on the weighted UniFrac distance metrics was performed to investigate the microbial community composition variations between different groups. PCoA analysis result showed that samples from the same group clustered together and separated from the other (Fig. 3). Similarities analysis demonstrated that microbial community compositions had significantly changed in the group L and H compared with group C, which means inflammation and colorectal cancer caused great variations in gut microbiota community compositions. (ANOSIM, group C vs group L vs group H, *r* = 0.79, *p*-value = 0.001; group C vs group L, *r* = 0.79, *p*-value = 0.03; group C vs group H: *r* = 0.98, *p*-value = 0.03). There were no significant variations between group L and H, suggesting little difference of gut microbiota community composition at the inflammation and CRC fully generated period (ANOSIM, group L vs group H, *r* = 0.40, *p* = 0.06).

### Bacterial taxonomic differences at the phylum level

**Fig. 4:**
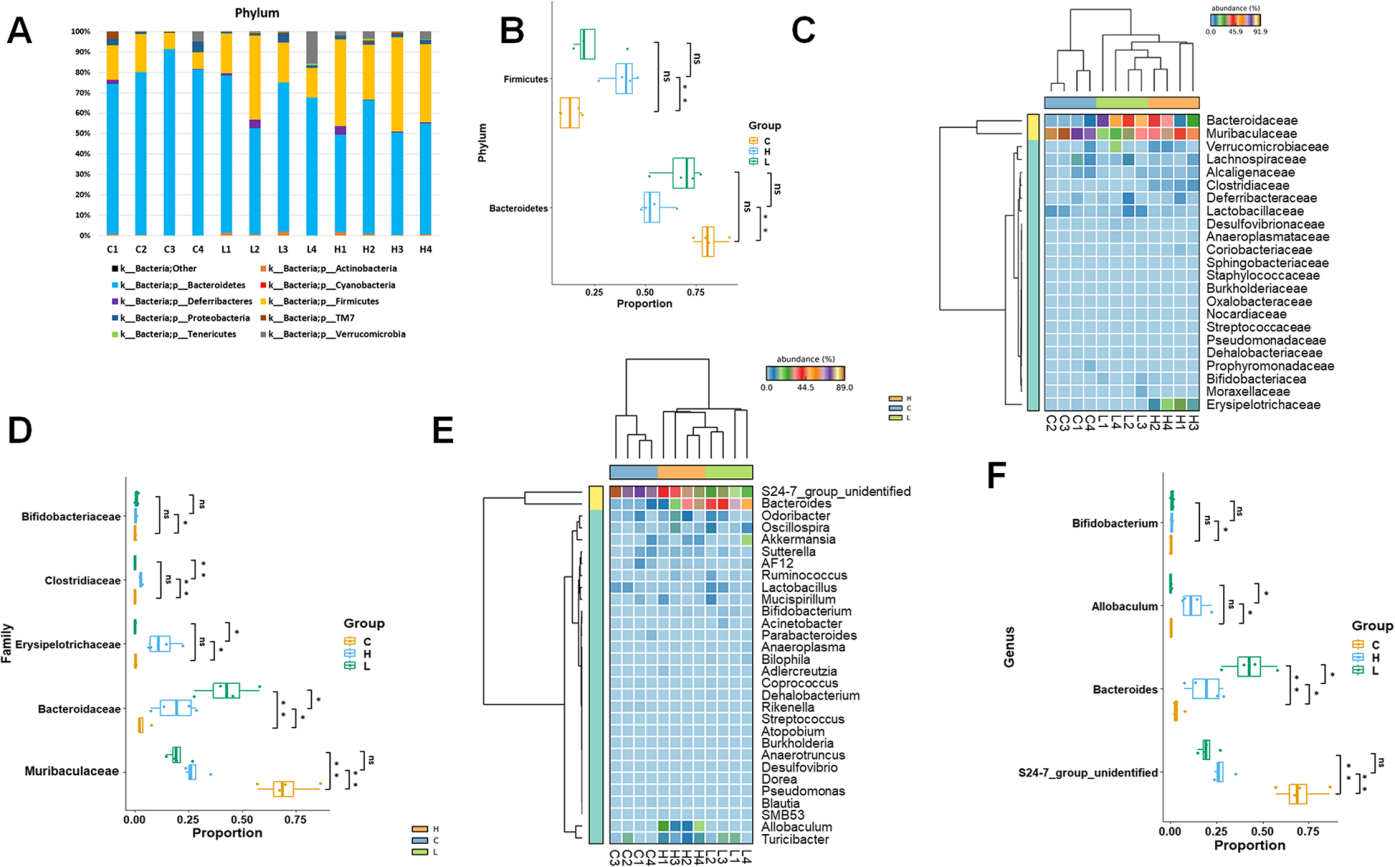
Changes in intestinal taxa in Group C, Group L and Group H. **A** Relative abundance of the microbial communities in Group C, Group L, Group H, revealed by the 16S rRNA gene at phylum level. The relative abundance is defined as a percentage of the total microbial sequences in a sample. **B** Boxplots of significantly changed floras at phylum level. **C** Heatmap of the 23 most abundant families contributes to characterize different CRC stages. **D** Boxplots of significantly changed floras at family level. **E** Heat map of the 37 most abundant genera contributes to characterize different CRC stages. **F** Boxplots of significantly changed floras at genus level. ns: *p* > 0.05,no significance;*: *p* < = 0.05;**: *p* < = 0.01

*Bacteroidetes* had highest abundance in each group at phylum level, and *Firmicutes* was the second most abundant phylum. Although *Bacteroidetes* and *Firmicutes* were the dominant bacteria in health and disease mice, the abundance of the dominant bacteria has changed during the colorectal cancer development (Fig. 4A). The average abundance of *Bacteroidetes* in group C, group L and group H were 81.5, 67.4, and 54.4%. The abundance of *Bacteroidetes* was decreased during the colorectal cancer formation. The average abundance of *Firmicutes* in group C, group L and group H were 13.0, 23.6 and 38.5%. The abundance of *Firmicutes* was increased during the colorectal cancer formation. Welch’s t-test was used to identify whether there were significant differences in microbial community composition due to CRC development (Fig. 4B). Significant variations in the abundance of *Bacteroidetes* (*p* = 0.002) and *Firmicutes* (*p* = 0.003) was observed between group C and H, suggesting that CRC formation greatly inhibited the growth of *Bacteroidetes* and was benefit to the growth of *Firmicutes*.

### Bacterial taxonomic differences at the family level

Welch’s t-test was used to find significantly changed bacteria due to the inflammation and colorectal cancer. A bunch of bacteria have significantly changed due to the colorectal cancer progress (Fig. 4D). The abundance of *Muribaculaceae* and *Bacteroidaceae* have significantly changed both in the initial inflammation stage and in the tumorigenesis stage of CRC compared with their abundance in health mice. *Bifidobacteriaceae*, *Clostridiaceae* and *Erysipelotrichaceae* were observed drastically responded to inflammation at beginning, however, they tend to back to their normal abundance later in the tumorigenesis stage of CRC. During the formation of colorectal cancer, *Clostridiaceae*, *Erysipelotrichaceae* and *Bacteroidaceae* in the initial stage of CRC were significantly different from those in the tumorigenesis stage of CRC. At family level, the dominant bacteria in group C were *Muribaculaceae* and *Bacteroidaceae*. Due to the inflammation and CRC development, the content of *Bacteroidaceae* was increased and became the most abundant microorganism in the intestinal tract (Fig. 4C). The average abundance of the significantly changed families were compared across different groups. The average abundance of *Muribaculaceae* in group C, group L and group H were 71.2, 21.4 and 30.8% respectively. *Bacteroidaceae*, its average abundance in group C, group L and H were 3.6, 42.7 and 18.8% respectively. *Erysipelotrichaceae*, in group C, its average abundance was 0.33%, in group L and H, its average abundance was 0.15 and 13.8%. *Clostridiaceae*, its average abundance was increased from 0.016% in group C to 3.05% in group H. The average abundance of *Bifidobacteriaceae* in group C, group L, and group H were 0.1, 0.8 and 0.6%. Overall, *Bacteroidaceae* showed its sensitivity among different stage of CRC development, and could be potentially used as a bacterial marker indicating the stages of colorectal cancer.

### Bacterial taxonomic differences at the genus level

At genus level, there were some important changes in the abundance of microorganisms (Fig. 4E, F). Genus *S24–7_group_unidifineted* belongs to family *Muribaculaceae*. *S24–7_group_unidifineted* had the highest average abundance in the feces samples. Its average abundance in group C, group L and group H was 70.2, 19.8 and 27.7%. Its abundance was significantly decreased due to inflammation and colorectal cancer. (group C vs group L: *p*-value = 5.16*10^− 5^, group C vs group H: *p*-value = 1.55*10^− 4^, group L vs group H: *p*-value = 0.03). *Bacteroides*, a subclass of *Bacteroidaceae*, its average abundance was 3.6% in group C, then increased to 42.7% in group L and 18.8% in group H. Welch’s t-test was used to evaluate whether there were significant differences in microorganisms’ abundance between different groups. The results showed that *Bacteroides* had significantly changed due to the inflammation and colorectal cancer. (group C vs group L: *p*-value = 3.21*10^− 3^, group C vs group H: *p*-value = 0.04, group L vs group H: p-value = 0.02). The average abundance of *Allobaculum* was 0.12% in group C and 0.11% in group L, which means inflammation has little effect on its abundance. However, after the CRC fully developed, its average abundance obviously increased to 12.6%. Result from Welch’s t-test showed that the abundance of *Allobaculum* had significantly changed in the CRC fully developed period (group C vs group H: *p*-value = 0.031, group L vs group H: *p*-value = 0.031). *Bifidobacterium* was initially affected by inflammation and later back to its normal abundance at CRC fully developed stage (group C vs group L: *p*-value = 0.04). Overall, *Bacteroides* was sensitive to both the inflammation and CRC, its abundance was statistically different at different stage of CRC development and could be potentially an indicator for CRC status. Both *Bacteroides* and *S24–7_group_unidifineted* were significantly changed after CRC fully developed, they could be used in CRC auxiliary diagnosis. The abundance of *Allobaculum* and *Bacteroides* were different in inflammation stage and colorectal cancer, they could be used to distinguish inflammation from colorectal cancer.

### Changes in predicted microbiota functional capacity after mice got colorectal inflammation and colorectal cancer

**Fig. 5:**
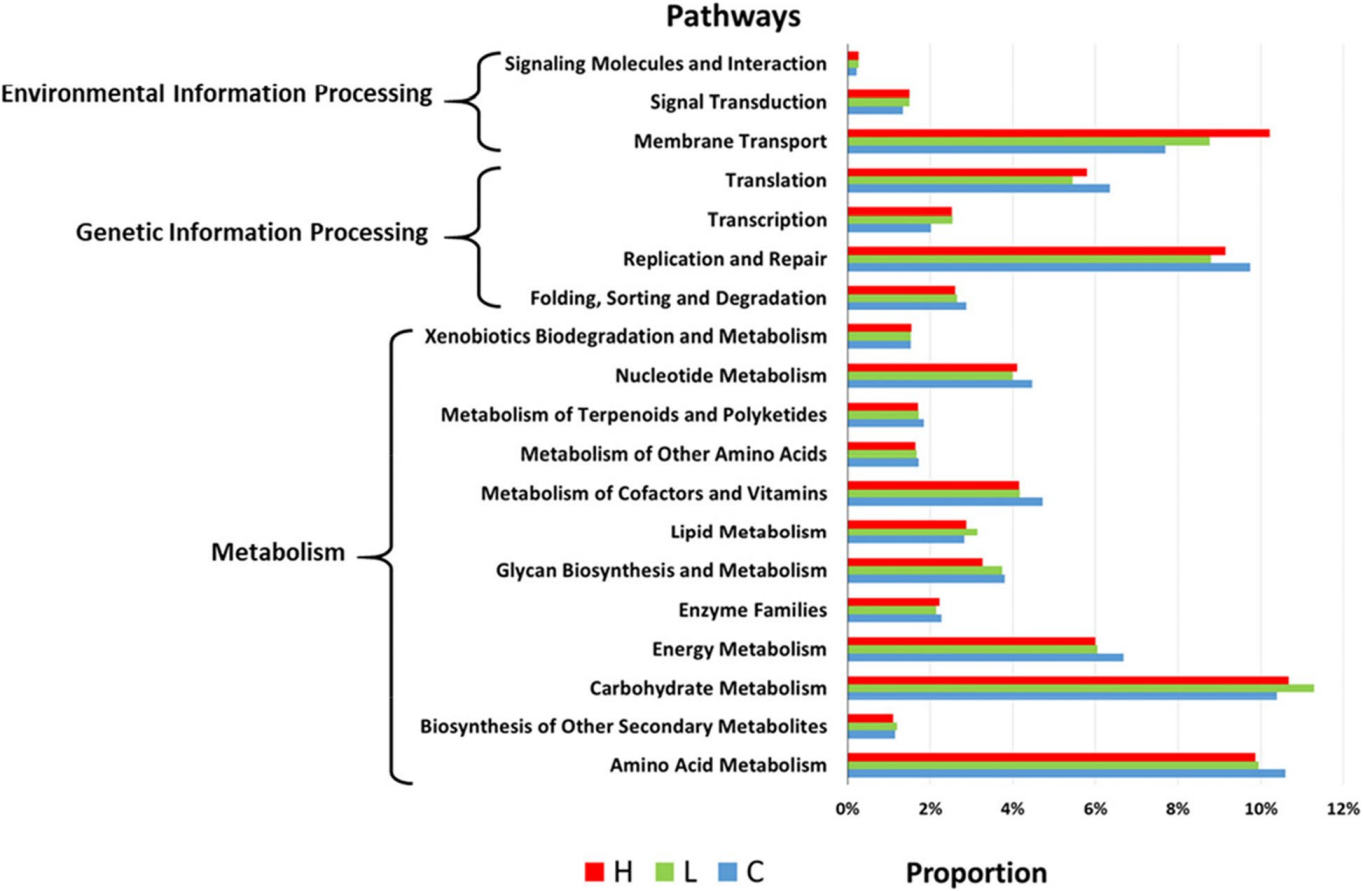
Phylogenetic investigation of communities by Reconstruction of Unobserved States (PICRUSt), predicting functional profile alteration of microbial communities enriched in Group C, Group L and Group H

The microbial community has significantly changed during the colorectal cancer development. Phylogenetic Investigation of Communities by Reconstruction of Unobserved States (PICRUSt) was used to predict the enriched metabolic pathway based on the enriched functional genes (Fig. 5). Compared with healthy mice, most of the metabolic pathways has changed in gut microbiota of the early inflammation stage and colorectal cancer, especially membrane transport, replication and repair, energy metabolism and amino acid metabolism. To evaluate the metabolic changes in the gut microbiota, Welch’s t-test was used to figure out the metabolic pathways that have significantly changed (q-value < 0.05), the result was listed in Table 2. In total, compared with healthy mice, in the early inflammation stage, transcription was significantly up regulated in gut microbiota. In both inflammation and CRC, metabolism of cofactors and vitamins, as well as energy metabolism were down regulated.

**Table 2:** Significantly changed pathways in different groups

Pathway	Compared groups	Alteration trend	Fold change	q-value
Energy Metabolism	L/C	**↓**	0.91	0.041
Enzyme Families	L/C	**↓**	0.94	0.041
Metabolism of Cofactors and Vitamins^a^	L/C	**↓**	0.89	0.029
Nervous System	L/C	**↑**	1.14	0.041
Poorly Characterized	L/C	**↑**	1.06	0.041
Transcription^a^	L/C	**↑**	1.27	0.041
Translation	L/C	**↓**	0.85	0.041
Amino Acid Metabolism	H/C	**↓**	0.93	0.031
Energy Metabolism	H/C	**↓**	0.90	0.031
Metabolism of Cofactors and Vitamins^a^	H/C	**↓**	0.88	0.031

^a^Significantly changed pathway related to colorectal cancer

### Associations of specific taxa and their interactions in the development of CRC

**Fig. 6:**
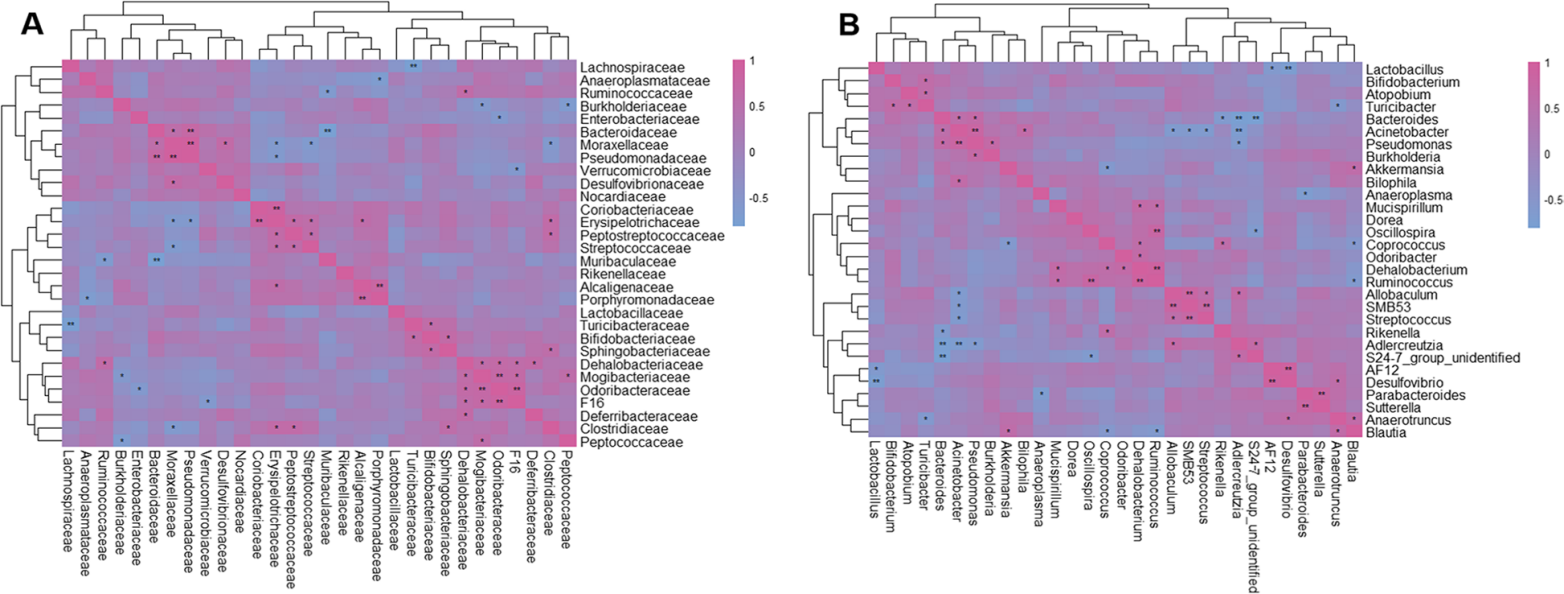
The correlation matrix of specific taxa. **A** Correlation analysis of the 30 most abundant families in Group C, Group L and Group H. **B** Correlation analysis of the 30 most abundant genera in Group C, Group L and Group H. *: 0.01 < *p* < = 0.05; **: *p* < = 0.01

In order to investigate the interactions of gut microbiota, spearman correlation analysis was conducted at family and genus level to draw the correlation matrix of 30 most abundant taxa in gut microbiota (*p* < 0.05) (Fig. 6A, B). We focus on the correlation of bacteria that have greatly changed due to the inflammation and colorectal cancer (*r* > 0.6 and *p* < 0.05). At family level, *Muribaculaceae* and *Bacteroidaceae* had a particularly strong excluding interaction (*r* = − 0.83, *p* = 1.7*10^− 3^). Meanwhile, *Bacteroidaceae* had a particularly strong positive interaction with *Pseudomonadaceae* (*r* = 0.73, *p* = 6.7*10^− 3^). Similarly, positively correlated bacterial patterns were observed. *Bifidobacteriaceae* was positively correlated to *Turicibacteraceae* and *Sphingobacteriaceae* (*r* = 0.62, *p* = 0.03; *r* = 0.68, *p* = 1.5*10^− 2^), *Erysipelotrichaceae* was positively correlated to *Alcaligenaceae, Streptococcaceae*, *Clostridiaceae*, *Coriobacteriaceae*, and *Peptostreptococcaceae* (*r* = 0.60, *p* = 0.04; *r* = 0.66, *p* = 0.04; *r* = 0.65, *p* = 0.02; *r* = 0.87, *p* = 3.0*10^− 4^; *r* = 0.67, *p* = 0.02). Besides, *Erysipelotrichaceae* was negatively correlated to *Moraxellaceae and Pseudomonadaceae* (*r* = − 0.62, *p* = 0.03; *r* = − 0.63, *p* = 0.03) At genus level, *Bacteroides* had a particularly strong excluding interaction with *S24–7_group_ unidentified* (*r* = − 0.83, *p* = 1.7*10^− 3^), *Adlercreutzia* (*r* = − 0.79, *p* = 3.6*10^− 3^) and *Rikenella* (*r* = − 0.65, *p* = 0.02). *Bacteroides* was strong positively correlated with *Pseudomonas* (*r* = 0.65, *p* = 0.02). *Allobaculum* had strong positively correlations with *Adlercreutzia* (*r* = 0.65, *p* = 0.02), SMB53 (*r* = 0.82, *p* = 1.2*10^− 3^), and negative correlations with *Acinetobacter* (*r* = − 0.65, *p* = 0.016). *Bifidobacterium* had a positively correlation with *Turicibacter*(*r* = 0.62, *p* = 0.03). *S24–7_group_ unidentified* was positively correlated with *Adlercreutzia* (*r* = 0.67, *p* = 0.02), it also had an excluding interaction with *Bacteroides* (*r* = − 0.83, *p* = 1.7*10^− 3^) and *Oscillospira* (*r* = − 0.66, *p* = 0.02).

## Discussion

The incidence of CRC is related to many factors. It is reported that the intestinal microbiota and its metabolism may be an important reason for the occurrence and development of colorectal cancer [14]. According to our research, the intestinal flora of mice changed significantly during the course of colorectal cancer. Although there was no significant difference in the α-diversity of gut microbiota in healthy, inflammation and colorectal cancer mice, the results of PCoA analysis and ANOSIM test showed significant difference of gut microbiota in β-diversity. The significant variations in β-diversity of gut microbiota due to inflammation or CRC were also observed in Liang, Xujun, et al.’s work [13]. In both Liang’s and our study, *Bacteroidetes* and *Firmicutes* were the dominant phyla. In our study, a variety of taxa in mice fecal samples changed significantly after the mice got inflammation and colorectal cancer, such as *Bacteroidetes* and *Firmicutes* at phylum level, *Muribaculaceae*, *Bacteroidaceae*, *Erysipelotrichaceae*, *Clostridiaceae* and *Bifidobacteriaceae* at family level, *S27_group_unidentified*, *Bacteroides*, *Allobaculum* and *Bifidobacterium* at genus level. The alternation of gut microbiota composition resulted in changed metabolic pathways (Table 2). Transcription as well as metabolism of cofactors and vitamins were pathways related to CRC. Transcription was significantly up regulated in gut microbiota of mice with inflammation suggesting an increased frequency of cell communications [15]. The excessive communications between gut microbiota and host induced by inflammation could increase the risk of CRC since gut microbiota produced carcinogenic toxins under inflammatory stress [16]. Metabolism of cofactors and vitamins was down regulated in gut microbiota of both inflammation and CRC. As we know intestinal microbes synthesized essential vitamins for host, problems in metabolism of cofactors and vitamins would certainly affected the gut health [17]. Thus, the transcription up-regulation together with metabolism of cofactors and vitamins down-regulation could facilitate the CRC formation.

Interactions between different types of intestinal bacteria was elucidated by correlation analysis. A variety of bacterial communities have mutual inhibition and mutual promotion effects. At genus level, *S24–7_group_unidentified* and *Bacteroides* were abundant in the intestinal tract and had strong excluding interaction. The highest abundance of *S24–7_group_unidentified* in healthy mice suggesting its great importance in maintaining a healthy microbial ecosystem. However, there was limited literature about *S24–7_group_unidentified* as it was newly discovered [16]. Thus, we look into the research on *Muribaculaceae* at family level. According to the existing research, the abundance of *Muribaculaceae* was affected by carbohydrates digesting [17]. Thus, we speculated that the energy metabolism of gut might have changed due to the CRC.

The abundances of *Bacteroides* and *Allobaculum* were significantly increased in intestinal tract of colorectal cancer mice which indicated that the two genera could be critical factors in promoting the pathological process of CRC. In our study, *Bacteroides* showed the same variation tendency as described in Liang, Xujun, et al.’s study and was most abundant in mice with inflammation, which suggests that the inflammation environment is benefit to the growth of *Bacteroides*. Increased *Bacteroides* content would inhibit the growth of *S24–7_group_unidentified*. Thus, inhibiting the growth of *Bacteroides* and promoting the growth of *S24–7_group_unidentified* could probably slow down the colorectal cancer formation.

## Conclusions

Our research characterized the composition of gut microbiota in CRC at early inflammation stage and fully developed stage. Intestinal flora had significantly changed in mice with inflammation or CRC compared with healthy mice. *S24–7_group_unidentified* and *Bacteroides* were the most abundant genus that have significantly changed in gut due to inflammation and CRC. A significant mutual inhibition between *S24–7_group_unidentified* and *Bacteroides* was found. Pathways enrichment analysis showed that metabolism of cofactors and vitamins, as well as energy metabolism have significantly changed in gut microbiota after the mice acquired inflammation and CRC.

### Materials and methods

#### Animal experiment and sample collection

Eight four-weeks-old male C57BL/6 mice were obtained from Nanjing Medical University (SPF grade, SCXK 2016–0002) and approved by the experimental animal administration committee of Jiangsu Simcere pharmaceutical Co., Ltd. (approval No. 011). General information of mice was provided in Additional file 1 table S2. After weighing, mice were evenly divided into two groups, control and experimental groups, each group had four replicates. After 1 week of acclimatization, mice in the control group were intraperitoneally injected with normal saline. While mice in the experimental group was intraperitoneally injected with AOM (12.5 mg/kg). After that, mice of experimental group would undergo intermittent oral administration of DSS. DSS (2.5%, w/w) was added to the drinking water of mice at week two, week five and week eight (Fig. 1A). While in the other weeks, mice were fed with normal drinking water. The DSS-water feeding circle will be conducted for 7 weeks. Mice in the control group were fed with normal drinking water during the entire experiment. Feces samples of experimental group mice were collected at the end of the first week’s DSS administration (L group) and the last 1 week’s DSS administration (H group). Feces samples in group C were collected from mice in experiment group before AOM and DSS treatment. Feces samples in group BC were collected from mice in control group at the same time as H group. At the time of feces samples collection, serum samples were also collected to evaluate the inflammation using MDA Assay Kit. MDA working solution was prepared according to manufacturer’s protocol. 0.6 mL working solution was added to 0.2 mL serum. The solution was incubated in 95 °C for 30 min and centrifuged at 10000 g for 10 min at 25 °C. The absorbance of the supernatant was detected at 532 nm and 600 nm. At the end of the experiment, the mice were all euthanized, and the colorectal tissues were analyzed by hematoxylin and eosin (HE) staining.

### 16S rRNA sequencing

The V3 to V4 region of the 16S rRNA gene was amplified with primer set 338F (50-ACTCCTACGGGAGGCAGCAG-30) and 806R (50- GGACTACHVGGGTWTCTAAT-30). Polymerase chain reaction (PCR) cycles were performed as follows: initial denaturation at 95 °C for 3 mins, followed by 27 cycles of heat and cooling, 95 °C for 30s, 55 °C for 30s, 72 °C for 45 s, and kept at 72 °C for 10 mins. The whole sequencing process was conducted by Shanghai Meiji Biomedical Technology Co., Ltd. (Shanghai, China) using an ABI GeneAmp® 9700 platform.

### Sequencing data analysis

Cutadapter (v1.10) was used to process our raw sequence reads [18]. FastQC (v0.11.9) was applied to evaluate data quality [19]. UCHIME2 was used to remove the chimera in the sequences [20], then UCLUST was used to cluster the sequences into operational taxonomic units (OTUs) with 97% similarity [21], the taxonomic classification was assigned by RDP classifier (v2.2) [22] against the Greengene database(v.13_8) [23].

### Statistical analysis

Qiime (v1.9.1) was used to perform α and β diversity analysis in order to show the microorganisms’ diversity and distribution [24]. Principle coordinate analysis (PCoA) was conducted using weighted UniFrac distance metrics. The dissimilarities between groups was illustrated by the analysis of similarities (ANOSIM). Boxplots and *p*-values were drawn and calculated by R.

To identify differentially changed microbiota during colorectal cancer development, paired t-test analysis of intestinal bacteria metagenomic profiles within two group was performed by statistical analysis of taxonomic and functional profiles (STAMP, v2) [25]. Bacteria that with fold change larger than 2 or smaller than 0.5 and p-value smaller than 0.05 were considered to have significant changes. Thirty mostly changed genera and families and their abundance were shown in heatmaps generated by STAMP (v2).

### Bacterial interactions and metagenomic functions

Spearman correlation analysis was used to calculate the correlation coefficients (r values) between microorganisms using most abundant 30 families and 30 genera. Significant correlations were defined as *r* > 0.8 or *r* < − 0.8 and *p*-value < 0.05.

Phylogenetic Investigation of Communities by Reconstruction of Unobserved States (PICRUSt,v1.1.0) [26] was used to predict the metabolic pathways and functional orthologs of microbial communities based on the Greengene database (v.13_5) and the Kyoto Encyclopedia of Genes and Genomes [27]. Welch’s t-test was used to figure out the metabolic pathways that have significantly changed (q-value < 0.05).

## Supplementary Information

**Additional file 1.**

## Data Availability

Sequencing data were deposited on NCBI under BioProject ID PRJNA718119.

## Abbreviations

PCoA: Principal coordinate analysis
ANOSIM: Analysis of similarities
CRC: Colorectal cancer
COX-2: Cyclooxygenase-2
NO: Nitric oxide
NOS-2: Nitric oxide synthase 2
AOM: Azoxymethane
DSS: Dextran sulfate sodium salt
MDA: Malondialdehyde
16S rRNA: Small subunit ribosomal RNA
OTU: Operational Taxonomic Unit
PICRUSt: Phylogenetic Investigation of Communities by Reconstruction of Unobserved States
HE: Hematoxylin and Eosin
