# Supplementary material for: Gut microbiota profiling variated during colorectal cancer development in mouse
Source: BMC Genomics. 2022 Dec 22;23(Suppl 4):848. doi: 10.1186/s12864-022-09008-3 (PMC9773433; doi:10.1186/s12864-022-09008-3)
Supplement: Supplementary file 1 — Additional file 1. [file 12864_2022_9008_MOESM1_ESM.docx]

**Gut microbiota profiling variated during colorectal cancer development in mouse**

Jingjing Liu, ^1, 2, +^ Wei Dong, ^1, +^ Jian Zhao ^1^, Jing Wu ^3^, Jinqiang Xia^2^, Shaofei Xie^2^, Xiaofeng Song^1, *^

^1^ Department of Biomedical Engineering, Nanjing University of Aeronautics and Astronautics, Nanjing 210016, China

^2^ The State Key Laboratory of Translational Medicine and Innovative Drug Development, Jiangsu Simcere pharmaceutical Co., Ltd., Nanjing 210016, China

^3^ School of Biomedical Engineering and Informatics, Nanjing Medical University, Nanjing, Jiangsu, 211166, China

Correspondence: xfsong@nuaa.edu.cn

^+^ The first 2 authors should be regarded as joint First Authors.

**Table S1.** Statistics of sequencing and α-diversity indexes of 16S rRNA. C: Group C, BC: Group BC.

| **Sample ID** | **Number of Reads** | **Observed OTUs** | **Chao1 Index** | **Shannon Index** | **Simpson Index** |
| --- | --- | --- | --- | --- | --- |
| **C1** | **18026** | **616.2** | **870.717** | **5.67** | **0.932** |
| **C2** | **24602** | **792.1** | **1148.002** | **6.225** | **0.958** |
| **C3** | **23776** | **762.3** | **1149.162** | **5.448** | **0.874** |
| **C4** | **18864** | **354.4** | **477.166** | **4.042** | **0.767** |
| **BC1** | **16254** | **699.0** | **911.379** | **5.86** | **0.914** |
| **BC2** | **28522** | **682.1** | **997.608** | **5.50** | **0.918** |
| **BC3** | **21147** | **815.9** | **1254.262** | **6.27** | **0.952** |
| **BC4** | **21531** | **478.8** | **681.862** | **5.08** | **0.886** |

**Table S2.** General information of mice.

| **Mouse ID** | **Gender** | **Age (week)** | **Strain** | **Body weight (g)** |
| --- | --- | --- | --- | --- |
| **M1** | **Male** | **4** | **C57BL/6** | **19.2** |
| **M2** | **Male** | **4** | **C57BL/6** | **20.5** |
| **M3** | **Male** | **4** | **C57BL/6** | **20.9** |
| **M4** | **Male** | **4** | **C57BL/6** | **19.8** |
| **M5** | **Male** | **4** | **C57BL/6** | **20.3** |
| **M6** | **Male** | **4** | **C57BL/6** | **20.9** |
| **M7** | **Male** | **4** | **C57BL/6** | **20.7** |
| **M8** | **Male** | **4** | **C57BL/6** | **21.2** |
| **M9** | **Male** | **4** | **C57BL/6** | **19.7** |
| **M10** | **Male** | **4** | **C57BL/6** | **19.5** |
| **M11** | **Male** | **4** | **C57BL/6** | **19.3** |
| **M12** | **Male** | **4** | **C57BL/6** | **19.4** |


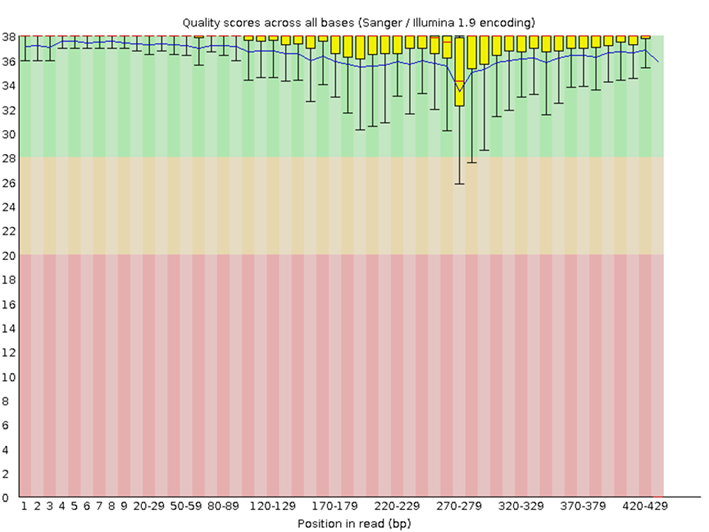


**Figure S1.** Distribution of quality scores at each position in the read across all reads.


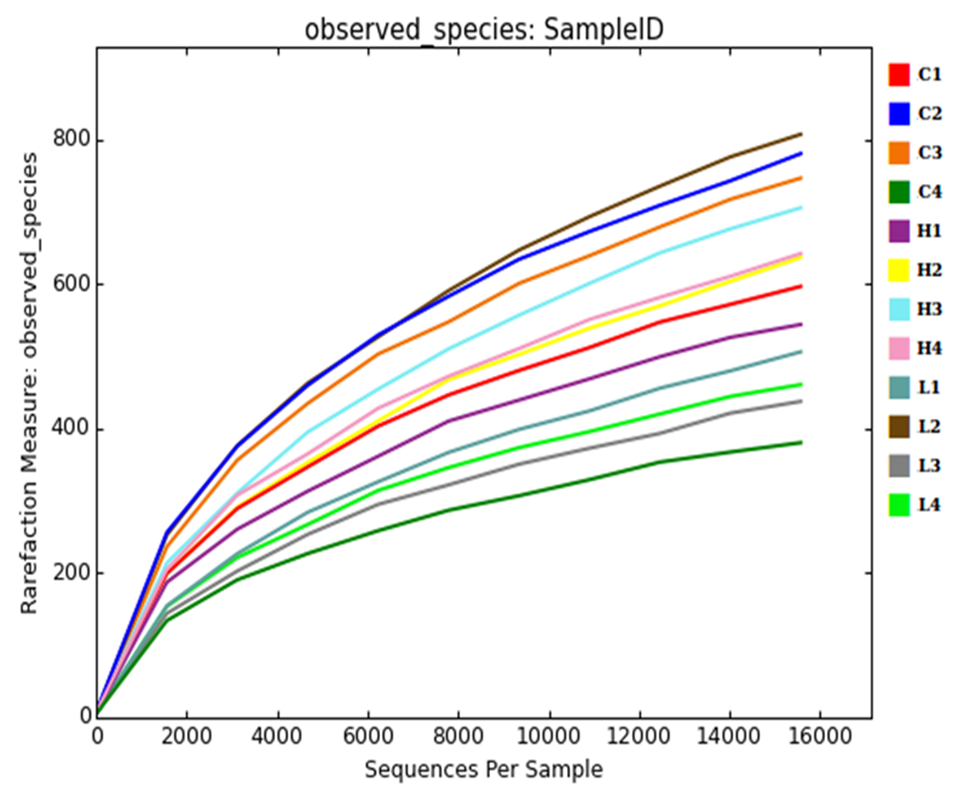


**Figure S2.** Rarefaction curve for each sample from group C, L and H based on the sequences of 16S rRNA genes**.**

**Figure S3.** Microbial α diversity in feces samples of Group C and Group BC. (A) Boxplots of Chao1 Richness Index. (B) Boxplots of Shannon Diversity Index. (C) Boxplots of Simpson Diversity Index. ns: p > 0.05,no significant difference.


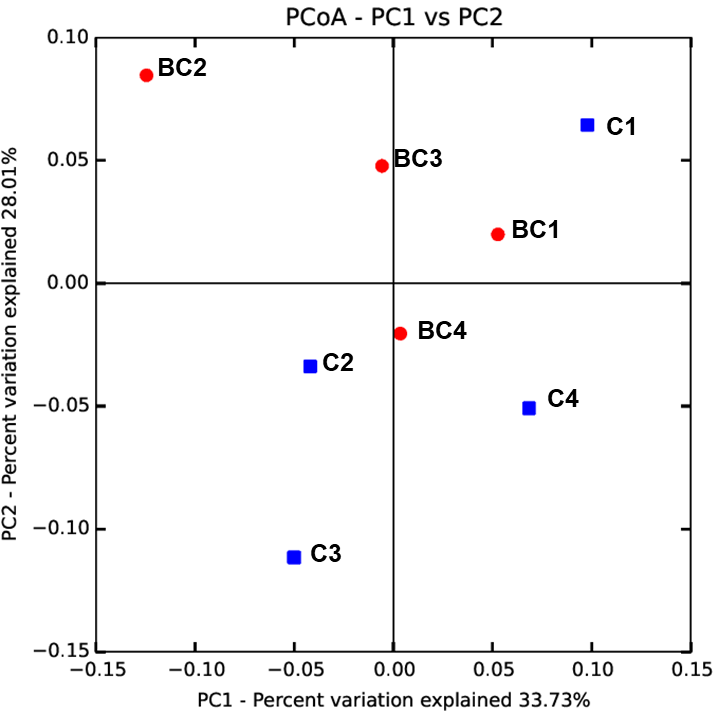


**Figure S4.** Microbial β diversity in feces samples of Group C and Group BC. PCoA plot of Group C and Group BC, there was no significant change in gut microbiota during mice growth.
